# Polycyclic aromatic hydrocarbon exposure and pediatric asthma in children: a case–control study

**DOI:** 10.1186/1476-069X-12-1

**Published:** 2013-01-03

**Authors:** Nasser M Al-Daghri, Majed S Alokail, Sherif H Abd-Alrahman, Hossam M Draz, Sobhy M Yakout, Mario Clerici

**Affiliations:** 1 Biomarkers Research Program, Biochemistry Department, College of Science, King Saud University, Riyadh 11451, Saudi Arabia; 2 Pesticides Residues and Environmental Pollution Dept., Central Agricultural Pesticide Laboratory, Agricultural Research Center, Giza 12618, Egypt; 3 Biochemistry Department, National Research Centre, Dokki, Cairo 12311, Egypt; 4 Department of Biomedical Sciences and Technology, Milano University Medical School, Segrate-Milano 20090, Italy and Fondazioe Don C Gnocchi, Milan, Italy

**Keywords:** PAHs, Environmental pollution, Childhood asthma, Biomarkers

## Abstract

**Background:**

Bronchial asthma is one of the most prevalent diseases in Arab children. Environmental pollution has been suggested to be considered causative of asthma, nasal symptoms and bronchitis in both children and adult. The objectives of this study were to evaluate the association between serum polycyclic aromatic hydrocarbons (PAHs) levels, asthma and allergic outcomes among Saudi children aged up to 15 yrs. We hypothesized that increased serum PAHs are associated with allergy, asthma, or respiratory symptoms.

**Methods:**

A total of 195 Saudi children (98 asthma pediatric patients and 97 healthy controls) were randomly selected from the Riyadh Cohort Study for inclusion. The diagnosis of Asthma was based on established pediatric diagnosis and medications taken.

**Results:**

Asthma related markers showed highly significant differences between children with and without asthma. Thus IgE, resistin and IL-4 were significantly increased (*p* 0.004, 0.001 and 0.003, respectively) in children with asthma compared with non-asthma control subjects. GMCSF, IFN-γ, IL-5, IL-8 and IL-10, on the other hand, were significantly decreased in children with asthma (*p* 0.003, 0.03, 0.001, 0.004 and 0.03, respectively). Strong associations between serum PAHs levels and biomarkers of childhood asthma were detected in Arabic children. Data confirmed the role of naphthalene, 4H-cyclobenta[def]phenanthrene, 1,2-benzanthracene, chrysene and benzo(e)acephenanthrylene in childhood asthma; levels of these PAHs were correlated with asthma related biomarkers including IgE, resistin, GMCSF and IFN-γ as well as IL-4, IL-5, IL-8 and IL-10 cytokines.

**Conclusions:**

This data highlight the pivotal role of specific PAHs in childhood asthma.

## Background

Asthma is one of the most common chronic diseases, that is characterized by variable airflow obstruction, inflammation of the airways, and bronchial hyper responsiveness. It affects nearly 14 million people worldwide and more than 2 million Saudis [1,2]. Asthma is the most frequent chronic illnesses in childhood [2-4]. It has an impact on patients, their families, and the community as a whole in terms of lost work and school days, poor quality of life and death cases [5]. Several potential determinants being responsible for development of atopy and asthma have been proposed, including lack of severe and repeated infections [6,7], obesity and lack of physical exercise [8], a small family size [9-11], changing dietary habits [12,13] and increased indoor allergen exposure [14,15].

Polycyclic aromatic hydrocarbons (PAHs) are produced during the incomplete combustion of organic material such as fuels, coal, wood, tobacco, and oil. Vehicle emissions are major sources of PAHs in urban areas [16,17]. Blood and urine PAHs levels are proposed as specific biomarkers of PAHs exposure via inhalation of polluted air and intake of certain foods (e.g. charcoal broiled meats and other smoked foods) [18]. Exposure to high levels of air pollutants has been associated with decreased lung function, asthma, nasal symptoms, bronchitis and sensitization to inhalant allergens in both children and adults [19-23]. These studies implicate traffic-related emissions, largely composed of diesel exhaust particles, trace metals, volatile organic com-pounds, nitrogen dioxide, particulate matter, and PAHs in the development of respiratory symptoms, asthma or the onset of allergies. Studies have shown that exposure to PAHs be associated with adverse respiratory health outcomes. Previous data reported that exposure to PAHs such as benz(a)anthracene, benzo(a)pyrene, benzo(b)fluoranthene, benzo(g,h,i)perylene, chrysene and dibenz(a,h)anthracene are associated with increased cough and wheeze at age 12 months, and breathing problems as well as development of asthma [24,25]. Notably, PAHs exposure was suggested to favor proallergic immunoglobulin IgE responses. Recently, the U.S. National Health and Nutrition Examination Survey (NHANES) (1999–2000, 2001–02) provided comprehensive descriptions of reference ranges for a larger panel of PAHs metabolites collected from a population of children and adults without suspected occupational exposures [20,22]. Higher levels were detected among children, suggesting that they may be at greater risk for adverse health effects. The objectives of this study were to evaluate the association between serum PAHs level with asthma among Saudi children up to 8 yrs. Our starting hypothesis being that increased serum PAHs are associated with allergy, asthma, or respiratory symptoms.

## Methods

### Study subjects

A total of 195 Saudis children aged 15 years old and below, (98 asthma pediatric patients and 97 healthy controls) were randomly selected from the Riyadh Cohort Study for inclusion. The parent or guardian of each child was asked to answer a questionnaire consisting of demographic information which included dietary questions, area of residence (near the factory, high-traffic area, etc.), presence of a smoker at home and other pertinent questions related to asthma. Asthma was based on established pediatric diagnosis and medications taken. Since most of the subjects were asymptomatic during the course of the study, parents were asked if the child had history of wheezing upon exhalation, frequent episodes of chest tightness and hyper-expansion of thorax with use of accessory muscles. Ethical approval was granted by the Ethics Committee of the College of Science Research Center, King Saud University, Riyadh, KSA.

### Anthropometrics

Anthropometry included height (rounded off to the nearest 0.5 cm) and weight (rounded off to the nearest 0.1 kg), which were measured using an appropriate international standard scale (Digital Person Scale; ADAM Equipment, Milford, CT, USA), as well as waist and hip circumference in centimeters, which were measured using a standard tape measure; and body mass index (BMI) was calculated as kg/m^2^. The Holtain Khan abdominal caliper by Holtain Ltd (Crymych, UK) was used to measure sagital abdominal diameter (SAD).

### Biochemical parameters

Fasting blood was collected by an assigned physician at their respective primary healthcare centers. Blood was drawn, centrifuged and processed on the same day. Both whole blood and serum were placed in plain polystyrene tubes. Serum was delivered to Biomarker Research Program (BRP) for storage at −20°C. Fasting serum glucose levels, and complete lipid profile (triglycerides, total cholesterol, high density lipoprotein [HDL]-cholesterol) were determined using a biochemical analyzer (Konelab, Espoo, Finland). Low density lipoprotein (LDL)-cholesterol was calculated using Friedman formula. This biochemical analyzer was calibrated routinely prior to the analysis of all serum samples using quality control samples provided by the manufacturer (Thermo-Fisher Scientific, Espoo, Finland). LINCOplex, human multiplex immunoassay kit based on Luminex 100 system platform (Luminex Corporation, Austin, TX, USA) was used for determination of three different panels by simultaneous detection of a great variety of cytokines, interleukins and immunoglobulines. The standard protocol for the process was followed. The concentrations of analyte in each sample were calculated with a five parameter model using Luminex IS software ver. 2.3.

### PAH analysis

A stock solution of 12 PAHs mixed standard solutions was prepared by dissolving 1 mg from each PAH in 100 ml acetonitrile. The series of PAHs mix standard (0.0, 0.5, 2.5, 5, 10, 50 and 100 ng ml-1) were prepared in acetonitrile for linearity. Calibration curves were generated by plotting peak area versus concentration. Each subject’s sample was analyzed for a suite of 12 PAHs as previously described [23]. Analytical determination was conducted by using liquid-liquid extraction followed by high performance liquid chromatography with fluorescence detector (HPLC-FLD). Standard calibration curve was presented excellent linearity, with good separation and repeatability. The limit of detection (LOD) was defined as the higher value of either the method blank LOD (three times standard deviation of method blank after subtracting the average blank), or the instrument LOD (signal >3 times the signal to noise ratio). The limit of quantification (LOQ) (signal >10 times the signal to noise ratio). The limits of detection were ranged from 1.2 to 4.0 ng ml^-1^ (0.001 ppm). The lowest possible standard on the calibration curve was accepted as the LOQ. The calibration curve and recovery validation study were all repeated three times (n = 3). Recovery and precision were estimated by using spiked blank matrix, samples were analysed in duplicate at five levels spread equally over the analytical range. The recoveries were calculated from the analytical signal as the ratio between found and expected expressed in %. The rate of recovery for all 12 PAHs were ranged from 86 to 106%.

### Statistical analysis

Data represented by mean ± standard deviation. Skewed data was either log or square root transformed. Non Gaussian variables were represented by median and inter quartile ranges. Two samples independent T–test was performed to compare control and asthma. Mann Whitney U test was performed to compare control and asthmatic, wherever variables don’t follow Gaussian distribution. *P* values <0.05 were considered statistically significant. Pearson’s correlation test was performed to examine various correlations. Analyses were performed with the SPSS-PC software, version 16.0 (SPSS Inc, Chicago, IL).

## Results

### Subjects characteristics

The epidemiological characterization of the 195 Saudis children included in the study (98 asthma pediatric patients and 97 non-asthma control subjects) is provided in Table 1. All children were comparable regards to BMI, waist, SAD, total cholesterol, glucose, triglycerides and LDL-cholesterol.

**Table 1:** Characterization of asthmatic children compared to non-asthma control subjects

**Parameters**	**Control**	**Asthma**	***P *value**
Age (years)	13.5 ± 4.2	13.7 ± 2.9	0.81
BMI (kg/m^2^)	21.1 ± 5.5	21.3 ± 6.6	0.81
Waist circumference (cm)	66.6 ± 15.1	70.7 ± 17.1	0.09
Hip circumference (cm)	82.1 ± 20.7	83.8 ± 19.9	0.54
SAD (cm)	17.0 ± 7.5	17.7 ± 19.6	0.53
Cholesterol (mmol/l)	4.3 ± 0.88	4.1 ± 0.72	0.07
Glucose (mmol/l)	5.3 ± 1.7	5.3 ± 1.7	0.90
HDL-Cholesterol (mmol/l)	1.04 ± 0.34	0.90 ± 0.31	0.008
Triglycerides (mmol/l)	1.1 ± 0.60	1.03 ± 0.41	0.59
LDL-Cholesterol (mmol/l)	2.8 ± 0.80	2.7 ± 0.67	0.21
IgE (pg/ml)	61.1 ± 4.3	123.1 ± 5.0	0.004
Resistin (pg/ml)	17.3 ± 1.3	24.5 ± 1.6	<0.001
GMCSF (pg/ml)	1.9 ± 0.10	1.0 ± 0.11	0.003
INF-gamma (pg/ml)	2.4 ± 0.74	1.6 ± 0.63	0.03
IL4 (pg/ml)	9.2 ± 1.4	18.5 ± 3.6	0.003
IL5 (pg/ml)	0.92 ± 0.20	0.52 ± 0.17	<0.001
IL8 (pg/ml)	12.4 ± 1.8	6.3 ± 1.2	0.004
IL10 (pg/ml)	12.1 ± 1.7	9.2 ± 1.4	0.03
Naphthalene (ng/ml)	10.7 ± 2.7	26.2 ± 2.5	<0.001
Anthracene (ng/ml)	239.5 ± 22.6	260.1 ± 52.3	0.79
Fluorene (ng/ml)	2.5 ± 0.75	3.6 ± 0.90	0.04
Phenanthrene (ng/ml)	9.3 ± 1.7	11.5 ± 1.9	0.13
4H-cyclobenta[def]phenanthrene (ng/ml)	6.2 ± 1.1	20.3 ± 2.1	<0.001
Pyrene (ng/ml)	0.67 ± 0.34	1.1 ± 0.50	0.04
Flouranthene (ng/ml)	26.9 ± 3.2	31.8 ± 3.4	0.60
1,2-benzanthracene (ng/ml)	0.37 ± 0.24	0.73 ± 0.36	0.01
Chrysene (ng/ml)	0.87 ± 0.19	1.8 ± 0.13	0.001
Benzo(*e*)pyrene (ng/ml)	1.6 ± 0.17	2.6 ± 0.20	0.32
Benzoacephenanthrylene (ng/ml)	1.7 ± 0.23	7.0 ± 0.90	0.006
Benzo(*a*)pyrene (ng/ml)	2.1 ± 0.80	4.8 ± 0.93	0.02

### Association between PAH and asthma markers

Not surprisingly, asthma related biochemical markers showed highly significant differences between children with asthma and non-asthma control subjects. Thus, compared to non-asthma control subjects significant increase in level of IgE (123.1 ± 5.0 vs. 61.1 ± 4.3, *p* = 0.004), resistin (24.5 ± 1.6 vs. 17.3 ± 1.3, *p* = 0.001) and IL-4 (18.5 ± 3.6 vs. 9.2 ± 1.4, *p =* 0.003) pg ml^-1^. While there was significant decrease in levels of GMCSF (1.0 ± 0.11 vs. 1.9 ± 0.1, *p* = 0.003), IFN-γ (1.6 ± 0.63 vs. 2.4 ± 0.74, *p* = 0.03), IL-5 (0.52 ± 0.17 vs. 0.92 ± 0.2, *p* = 0.001), IL-8 (6.3 ± 1.2 vs. 12.4 ± 1.8, *p* = 0.004) and IL-10 (9.2 ± 1.4 vs. 12.1 ± 1.7, *p* = 0.03) pg ml^-1^.

Quantification of PAHs showed the presence of significant difference between children with asthma and non-asthma control subjects in levels of naphthalene (26.2 ± 2.5 vs. 10.7 ± 2.7, *p* 0.001), fluorene (3.6 ± 0.90 vs. 2.5 ± 0.75, *p* 0.04), 4H-cyclobenta[def]phenanthrene (20.3 ± 2.1 vs. 6.2 ± 1.1, *p* = 0.001), pyrene (1.1 ± 0.50 vs. 0.67 ± 0.34, *p* = 0.04), 1,2-benzanthracene (0.73 ± 0.36 vs. 0.37 ± 0.24, *p* = 0.01), chrysene (1.8 ± 0.13 vs. 0.87 ± 0.19, *p* = 0.001), benzoacephenanthrylene (7.0 ± 0.90 vs. 1.7 ± 0.23, *p* = 0.006) and benzo(a)pyrene (4.8 ± 0.93 vs. 2.1 ± 0.80, *p* = 0.02) ng ml^-1^. In contrast with these results, no differences were detected between the two groups in the levels of anthracene, phenanthrene, fluoranthene and benzo(e)pyrene.

To examine the role of PAHs in asthma, we analysed the association between PAHs and markers of asthma (Table 2). Our results clearly showed strong correlation between serum PAHs levels and asthma related biomarkers in Arab children. Thus, serum naphthalene was positively associated with IgE (r = 0.18, *p* = 0.03), IL-4 (r = 0.54, *p* =0.001) and resistin (r = 0.19, *p* = 0.02) and negatively associated with IL-5 (r = −0.18, *p* = 0.03). 4H-cyclobenta[def]phenanthrene was positively associated with IgE (r = 0.18, *p* = 0.03), IL-4 (r = 0.54, *p* = 0.001) and negatively associated with IL-10 (r = −0.23, p = 0.02). 1,2-benzanthracene was positively associated with IgE (r = 0.27, *p* = 0.006), resistin (r = 0.34, *p* =0.001) and negatively associated with IL-10 (r = −0.21, *p* = 0.03). Benzo(e)acephenanthrylene was positively associated with IL-4 (r = 0.45, *p* = 0.001), resistin (r = 0.28, *p* = 0.004) and negatively associated with IL-5 (r = −0.26, *p* = 0.009), IL-10 (r = −0.28, *p* = 0.005). Benzo(a)pyrene was positively associated with resistin (r = 0.37, *p* = 0.001) and negatively associated with IL-10 (r = −0.26, *p* = 0.009).

**Table 2:** Correlation between asthma related biomarkers and serum PAH Levels

	**Resistin**	**IFN-γ**	**IL-4**	**IL-5**	**IL-8**	**IL-10**	**IgE**
**Naphthalene**	0.19*	−0.05	0.54**	0.18*	−0.08	−0.11	0.18*
**Anthracene**	0.02	−0.004	0.05	−0.04	−0.03	−0.07	0.05
**Flourene**	−0.07	−0.08	−0.07	−0.14	−0.03	−0.003	−0.03
**Phenantherene**	0.11	−0.10	0.25**	0.06	0.09	−0.06	−0.14
**4H-cyclobenta[def] phenanthrene**	0.15	−0.18	0.78**	−0.12	−0.17	−0.23*	0.20*
**Pyrene**	0.008	−0.07	−0.09	0.08	−0.21*	0.06	0.21*
**Flouranthene**	0.26**	−0.19*	0.17	0.03	−0.07	0.10	−0.006
**1,2-benzanthracene**	0.34**	0.05	−0.14	0.002	−0.06	−0.21*	0.27**
**Chrysene**	0.03	0.001	0.08	0.31**	−0.23*	0.06	0.16
**Benzo(e)pyrene**	0.13	0.007	0.32**	−0.17	0.02	−0.17	−0.03
**Benzo(e)acephenanthrylene**	0.28**	−0.10	0.45**	0.26**	−0.01	−0.28**	0.08
**Benzo(a)pyrene**	0.37**	0.11	−0.17	0.07	0.01	−0.26**	0.06

(*): significant at *P* ≤ 0.05.

(**): significant at *P* ≤ 0.01.

## Discussion

Our objectives were to analyze serum PAHs levels in children with asthma, establishing possible correlations between such levels and asthma-related biomarkers. Data herein show that different levels of PAHs are detected in asthmatic compared to non-asthma control subjects. PAHs levels were significantly increased in asthmatic individuals compared to non-asthma control subjects. Differences were observed in PAHs levels, depending on the individual PAH, but most commonly for naphthalene, 4H-cyclobenta[*def*]phenanthrene, 1,2-benzanthracene, chrysene, Benzo(*e*)acephenanthrylene. Previous studies considered naphthalene to be a more specific indicator of exposure to airborne PAH exposure [26,27]. PAHs level detected are likely to reflect differences attributable to the ubiquitous traffic-related and other sources of air pollution in the urban environment of the studied cohort. These findings also could reflect differences in dietary intake [28]. Regardless, they may be indicators of greater potential risk for the later development of PAH-associated diseases.

Mechanisms underlying the differences in PAHs levels may be multifactorial. These may be impacted by dietary preferences or lifestyle behaviors not measured in this study which might explain such changes. Additionally, genetic factors may impact on the metabolism of these compounds. Some of these possibilities have also been suggested by the NHANES report [20].

The present study showed positive associations between IgE and PAHs (naphthalene, 4H-cyclobenta[def]phenanthrene, 1,2-benzanthracene). The associations between PAHs levels and IgE levels reported in other studies indeed suggest that exposure to traffic-related air pollution results in an up-regulation of allergic immune responses [29-31]. *In vitro* studies have demonstrated that PAH-enhance IgE production via their effects on tensile B cells [32]. Exposure to pyrene has been associated with up-regulation of the IL-4 promoter [33]. In addition, different PAHs pyrene, benzo(a)-pyrene, anthracene, phenanthrene and fluoranthene has been shown to induce IgE production, T helper 2 cytokine productions in both rodent and studies highlighting the cellular impact of PAHs on metabolism and the immune response [34-36].

## Conclusion

Our results show that serum PAHs level is correlated with multiple asthma related biomarkers in children. Results herein also support the importance of Naphthalene, 4H-cyclobenta[def]phenanthrene, 1,2-benzanthracene, Chrysene and Benzo(e)acephenanthrylene in childhood asthma and clearly indicate that the plasma concentration of these compounds correlate with asthma related biomarkers including IgE, resistin, GMCSF, IFN-γ, IL-4, IL-5, IL-8 and IL-10.

## Abbreviations

PAHs: Polycyclic aromatic hydrocarbons; BMI: Body mass index; LDL: Low density lipoprotein; HDL: High density lipoprotein; SAD: Sagittal abdominal diameter.

## Competing interests

The authors declared no competing interest with respect to the authorship and/or publication of this article.

## Authors’ contributions

NMAD, MSA and SHAA designed and initiated the current study. NMAD, SHAA and MC were responsible for collecting the samples and the interview data. SHAA and HMD coordinated the current study. SHAA was responsible for the PAHs analysis using HPLC-FLD. HMD and SMY were responsible for the biochemical analyses. SHAA and HMD were responsible for writing the draft version of manuscript. NMAD, MSA, SMY and MC were responsible for reviewing the draft version of manuscript. All authors commented on and approved the final manuscript.

## References

[B1] Weiss KB Shannon JJ Sadowski LS Sharp LK Curtis L Lyttle CS Kumar R Shalowitz MU Weiselberg L Catrambone CD Evans A Kee R Jon Miller J Kimmel L Grammer L The burden of asthma in the Chicago community fifteen years after the availability of national asthma guidelines: the design and initial results from the CHIRAH study Contemp Clin Trials 2009 30 246 255 10.1016/j.cct.2009.01.006 19470314 PMC3509188

[B2] Al Frayh AR Shakoor Z Gad El Rab MO Hasnain SM Increased prevalence of asthma in Saudi Arabia Ann Allergy Asthma Immunol 2001 86 3 292 296 10.1016/S1081-1206(10)63301-7 11289327

[B3] Essam HJ Nasser AE Association between b + 252 tumour necrosis factor polymorphism and asthma in western Saudi children Saudi Journal of Biological Sciences 2011 18 107 111 10.1016/j.sjbs.2010.10.006 23961111 PMC3730738

[B4] Hijazi N Abalkhail B Seaton A Asthma and respiratory symptoms in urban and rural Saudi Arabia Eur Respir J 1998 12 41 44 10.1183/09031936.98.12010041 9701412

[B5] Stewart WF Ricci JA Chee E Morganstein D Lost productive work time costs from health conditions in the United States: results from the American Productivity Audit J Occup Environ Med 2003 45 1234 1246 10.1097/01.jom.0000099999.27348.78 14665809

[B6] Shaheen SO Aaby P Hall AJ Barker DJ Heyes CB Shiell AW Goudiaby A Measles and atopy in Guinea-Bissau Lancet 1996 347 1792 1796 10.1016/S0140-6736(96)91617-7 8667923

[B7] Shirakawa T Enomoto T Shimazu S Hopkin JM The inverse association between tuberculin responses and atopic disorder Science 1997 275 77 79 10.1126/science.275.5296.77 8974396

[B8] Shaheen SO Obesity and asthma: cause for concern? Clin Exp Allergy 1999 29 3 291 293 10.1046/j.1365-2222.1999.00516.x 10202333

[B9] Bodner C Godden D Seaton A Family size, childhood infections and atopic diseases. The Aberdeen WHEASE Group Thorax 1998 53 28 32 10.1136/thx.53.1.28 9577518 PMC1758702

[B10] Jarvis D Chinn S Luczynska C Burney P The association of family size with atopy and atopic disease Clin Exp Allergy 1997 27 240 245 10.1111/j.1365-2222.1997.tb00701.x 9088649

[B11] Strachan DP Hay fever, hygiene, and household size BMJ 1989 299 6710 1259 1260 10.1136/bmj.299.6710.1259 2513902 PMC1838109

[B12] Hodge L Salome C Hughes J Effect of Dietary intake of omega-3-fatty acids and omega-6-fatty acids on severity of asthma in children Eur Respir J 1997 II 361 365 10.1183/09031936.98.11020361 9551739

[B13] Weiland SK von Mutius E Husing A Asher MI Intake of trans fatty acids and prevalence of childhood asthma and allergies in Europe. ISAAC Steering Committee Lancet 1999 353 2040 2041 10.1016/S0140-6736(99)01609-8 10376626

[B14] Peat JK Li J Reversing the trend: reducing the prevalence of asthma J Allergy Clin Immunol 1999 103 1 10 10.1016/S0091-6749(99)70517-8 9893177

[B15] Sporik R Holgate ST Platts-Mills TA Cogswell JJ Exposure to house-dust mite allergen (Der p I) and the development of asthma in childhood. A prospective study N Engl J Med 1990 323 502 507 10.1056/NEJM199008233230802 2377175

[B16] Harrison RM Johnston WR Deposition fluxes of lead, cadmium, copper and polynuclear aromatic hydrocarbons (PAH) on the verges of a major highway Sci Total Environ 1985 46 121 135 10.1016/0048-9697(85)90289-X 2417311

[B17] Vardoulakis S Chalabi Z Fletcher T Grundy C Leonardi GS Impact and uncertainty of a traffic management intervention: population exposure to polycyclic aromatic hydrocarbons Sci Total Environ 2008 394 244 251 10.1016/j.scitotenv.2008.01.037 18295302

[B18] Brandt HC Watson WP Monitoring human occupational and environmental exposures to polycyclic aromatic compounds Ann Occup Hyg 2003 47 349 378 10.1093/annhyg/meg052 12855487

[B19] Vyskocil A Fiala Z Chenier VV Krajak L Ettlerova E Bukac J Viau C Emminger S Assessment of multipathway exposure of small children to PAH Environ Toxicol Pharmacol 2000 8 111 118 10.1016/S1382-6689(00)00032-6 10867370

[B20] Li Z Sandau CD Romanoff LC Caudill SP Sjodin A Needham LL Patterson DG Jr Concentration and profile of 22 urinary polycyclic aromatic hydrocarbon metabolites in the US population Environ Res 2008 107 320 331 10.1016/j.envres.2008.01.013 18313659

[B21] Perera FP Rauh V Tsai WY Kinney P Camann D Barr D Bernert T Garfinkel R Tu YH Diaz D Dietrich J Whyatt RM Effects of transplacental exposure to environmental pollutants on birth outcomes in a multiethnic population Environ Health Perspect 2003 111 201 205 12573906 10.1289/ehp.5742 PMC1241351

[B22] Grainger J Huang W Patterson DG Jr Turner WE Pirkle J Caudill SP Wang RY Needham LL Sampson EJ Reference range levels of polycyclic aromatic hydrocarbons in the US population by measurement of urinary monohydroxy metabolites Environ Res 2006 100 394 423 10.1016/j.envres.2005.06.004 16225859

[B23] Al-Daghri NM Serum polycyclic aromatic hydrocarbons among children with and without asthma: correlation to environmental and dietary factors Int J Occup Med Environ Health 2008 21 211 217 18980880 10.2478/v10001-008-0021-0

[B24] Miller RL Garfinkel R Horton M Camann D Perera FP Whyatt RM Kinney PL Polycyclic aromatic hydrocarbons, environmental tobacco smoke, and respiratory symptoms in an inner-city birth cohort Chest 2004 126 1071 1078 10.1378/chest.126.4.1071 15486366 PMC2223076

[B25] Jedrychowski W Galas A Pac A Flak E Camman D Rauh V Perera F Prenatal ambient air exposure to polycyclic aromatic hydrocarbons and the occurrence of respiratory symptoms over the first year of life Eur J Epidemiol 2005 20 775 782 10.1007/s10654-005-1048-1 16170661

[B26] Kang JW Cho SH Kim H Lee CH Correlation of urinary 1-hydroxypyrene and 2-naphthol with total suspended particulates in ambient air in municipal middle-school students in Korea Arch Environ Health 2002 57 377 382 10.1080/00039890209601425 12530608

[B27] Yang M Koga M Katoh T Kawamoto T A study for the proper application of urinary naphthols, new biomarkers for airborne polycyclic aromatic hydrocarbons Arch Environ Contam Toxicol 1999 36 99 108 10.1007/s002449900447 9828267

[B28] Falco G Domingo JL Llobet JM Teixido A Casas C Muller L Polycyclic aromatic hydrocarbons in foods: human exposure through the diet in Catalonia Spain. J Food Prot 2003 66 2325 2331 10.4315/0362-028x-66.12.2325 14672232

[B29] Morgenstern V Zutavern A Cyrys J Brockow I Koletzko S Kramer U Behrendt H Herbarth O von Berg A Bauer CP Wichmann HE Heinrich J Atopic diseases, allergic sensitization, and exposure to traffic-related air pollution in children Am J Respir Crit Care Med 2008 177 1331 1337 10.1164/rccm.200701-036OC 18337595

[B30] Nel AE Diaz-Sanchez D Ng D Hiura T Saxon A Enhancement of allergic inflammation by the interaction between diesel exhaust particles and the immune system J Allergy Clin Immunol 1998 102 539 554 10.1016/S0091-6749(98)70269-6 9802360

[B31] Wyler C Braun-Fahrlander C Kunzli N Schindler C Ackermann-Liebrich U Perruchoud AP Leuenberger P Wuthrich B Exposure to motor vehicle traffic and allergic sensitization. The Swiss Study on Air Pollution and Lung Diseases in Adults (SAPALDIA) Team Epidemiology 2000 11 450 456 10.1097/00001648-200007000-00015 10874554

[B32] Takenaka H Zhang K Diaz-Sanchez D Tsien A Saxon A Enhanced human IgE production results from exposure to the aromatic hydrocarbons from diesel exhaust: direct effects on B-cell IgE production J Allergy Clin Immunol 1995 95 103 115 10.1016/S0091-6749(95)70158-3 7529782

[B33] Bommel H Li-Weber M Serfling E Duschl A The environmental pollutant pyrene induces the production of IL-4 J Allergy Clin Immunol 2000 105 796 802 10.1067/mai.2000.105124 10756232

[B34] Kanoh T Suzuki T Ishimori M Ikeda S Ohasawa M Ohkuni H Tunetoshi Y Adjuvant activities of pyrene, anthracene, fluoranthene and benzo(a)pyrene in production of anti-IgE antibody to Japanese cedar pollen allergen in mice J Clin Lab Immunol 1996 48 133 147 9819666

[B35] Takano H Yoshikawa T Ichinose T Miyabara Y Imaoka K Sagai M Diesel exhaust particles enhance antigen-induced airway inflammation and local cytokine expression in mice Am J Respir Crit Care Med 1997 156 36 42 9230723 10.1164/ajrccm.156.1.9610054

[B36] Fujimaki H Shiraishi F Aoki Y Saneyoshi K Modulated cytokine production from cervical lymph node cells treated with B[a]P and PCB Chemosphere 1997 34 1487 1493 10.1016/S0045-6535(97)00445-1 9134681

